# Great cities look small

**DOI:** 10.1098/rsif.2015.0315

**Published:** 2015-08-06

**Authors:** Aaron Sim, Sophia N. Yaliraki, Mauricio Barahona, Michael P. H. Stumpf

**Affiliations:** 1 Department of Mathematics, Imperial College London, London SW7 2AZ, UK; 2 Department of Chemistry, Imperial College London, London SW7 2AZ, UK; 3 Department of Life Sciences, Imperial College London, London SW7 2AZ, UK

**Keywords:** infrastructure, population models, social networks

## Abstract

Great cities connect people; failed cities isolate people. Despite the fundamental importance of physical, face-to-face social ties in the functioning of cities, these connectivity networks are not explicitly observed in their entirety. Attempts at estimating them often rely on unrealistic over-simplifications such as the assumption of spatial homogeneity. Here we propose a mathematical model of human interactions in terms of a local strategy of maximizing the number of beneficial connections attainable under the constraint of limited individual travelling-time budgets. By incorporating census and openly available online multi-modal transport data, we are able to characterize the connectivity of geometrically and topologically complex cities. Beyond providing a candidate measure of greatness, this model allows one to quantify and assess the impact of transport developments, population growth, and other infrastructure and demographic changes on a city. Supported by validations of gross domestic product and human immunodeficiency virus infection rates across US metropolitan areas, we illustrate the effect of changes in local and city-wide connectivities by considering the economic impact of two contemporary inter- and intra-city transport developments in the UK: *High Speed 2* and *London Crossrail*. This derivation of the model suggests that the scaling of different urban indicators with population size has an explicitly mechanistic origin.

## 1. Introduction

Can the greatness of a city be quantified? The city of Nineveh, capital of the Neo-Assyrian empire of 911–627 BC, was once described as ‘an exceedingly great city, three days' journey in breadth’ [1]. Today, a city described as such would more likely be dismissed as an urban sprawl let down by an inefficient transport infrastructure. Without reference to travelling-time constraints, size is clearly not a sufficient measure of greatness—just like rank and title can be poor predictors of influence in social networks [2,3]. Of the many candidates [4,5], the simplest objective measure of success is, possibly, the extent to which a city fulfils its primary purpose of maximizing the number of face-to-face, opportunity-spawning, interactions between its inhabitants [6]. From the rise of the Medici in fifteenth century Florence to the prestige of an efficient transport system in a twenty-first century metropolis, this *connectivity* is synonymous with both the eminence of individuals and the success of whole cities [7–11].

Measuring this connectivity, however, is not straightforward. Despite the success of social theory and experiments in much smaller contexts [12–14], the number of face-to-face social ties in a city, unlike secondary socio-economic indicators, remains poorly estimated. Beneath the reductionist representation of cities as featureless groups of individuals lies a forbidding, real-world diversity [7], including widely differing population sizes (approx. 10^3^–10^7^), distributions (uniform, polycentric [15]), topologies and geometries, the latter covering both geography (boundaries, natural features) and the different modalities of transport infrastructure (rail networks, traffic) [16]. In addition, cultural and activity-specific behavioural difference (e.g. travelling-time tolerances) is a complicating factor in theories of urban human interactions.

A typical strategy is to ignore this heterogeneity in favour of simple summary statistics like population size [17], density [18], or even congestion sensitivity [4] or a global fractional dimensionality [19]. However, comparing cities that differ significantly on any of the excluded characteristics is then simply not possible with these models. Of particular significance to city planners, such models are, for the same reasons, unsuitable for assessing the impact of complex infrastructure or demographic changes.

The parsimony of such approaches is, nevertheless, not without merit. Most notably, there is an apparent common scaling with respect to population size across a wide range of urban indicators [20]. However, this empirical scaling is similar but not identical across indicators, both in the scaling exponent *β* and level of statistical support (e.g. US 2002 new AIDS cases exhibit a power law against population with an exponent *β* = 1.23 and correlation coefficient Adj-*R*^2^ = 0.76 while private R&D employment has *β* = 1.34 with Adj-*R*^2^ = 0.92) [17]. Furthermore, power-law relationships can also arise by chance or as statistical artefacts, and even if supported by data they are largely descriptive and do not constitute constructive mechanistic narratives [21,22]. Indeed, recent attempts (such as in [19,23,24]) to lift this science of cities above the level of descriptive statistics reflect a growing desire for more generative and explanatory models.

A major step in this direction was taken by Pan *et al.* in [18] where the observations behind the super-linear scaling relations were shown to be entirely consistent with—and actually better modelled by—the more fundamental assumption that the probability of social-tie formation between two individuals is inversely proportional to the number of people in closer proximity. Despite the arbitrary nature of the probability ansatz, this elegant reduction of purely phenomenological power-law statistical observations to a statement about the likelihood of interactions between pairs of individuals suggests the existence of an underlying set of behavioural principles governing the formation of the network of social ties in a city.

In this paper we propose one such set of rules. These rules are ‘parameter-free’ in the sense that they do not depend on any arbitrary functional assumptions beyond several intuitive statements on human behaviour. We build from them a model for real-world deliberate (as opposed to accidental or serendipitous) social interactions derived solely in terms of this set of agent-driven principles and is, therefore, by design, truly *mechanistic*. In particular, via our derivation from first principles, we show how the probability of social-tie formation originally proposed in [25] can be viewed as an emergent consequence of these more fundamental and, crucially, mechanistic principles. On a practical side, the model readily incorporates available detailed demographic, transportation and economic data, thereby providing a tool for the *a priori* assessment of the effectiveness of planned infrastructure measures.

## 2. A model of deliberate social ties

### 2.1. Modelling principles

We start with four principles, the justification for and mathematical implications of which we will shortly unpack:

(1) Individuals are characterized by a set of attributes (*heterogeneity*).
(2) For each attribute, individuals seek out social ties only with others who have higher attribute values (*utility optimization*).
(3) Individuals have a set of attribute-specific travelling-time budgets *τ*_max_ (*resource constraints*).
(4) A directed tie is formed only if there are no closer and better opportunities in the proximity of the seeker (*intervening opportunities*).

#### 2.1.1. Heterogeneity

The first principle is a nod to the variety of city life. Besides a multitude of attributes—from objective (e.g. wealth) to subjective (e.g. beauty), from beneficial (e.g. artistic skills) to harmful (e.g. criminality)—there exists a spectrum of skills and levels in those attributes across the population. To represent this heterogeneous set of attributes, we define a set of non-identically distributed random variables2.1
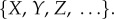


Each set of realizations {*x*, *y*, *z*, … } then represents an individual's set of abilities and scores in the corresponding attributes.

#### 2.1.2. Utility optimization

The second principle is a statement of human endeavour, whereby one seeks to build beneficial ties. It is simply a variation on the theory of rational choice where individuals are deemed to act in their own perceived best interest [26]. For a given attribute *Z*, we express this necessary condition for a directed social tie from person *i* to person *j* as2.2
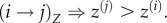


#### 2.1.3. Resource constraints

The third principle reflects the finite nature of individual resources by adopting the concept of the travelling-time budget *τ*_max_, that is the maximum amount of time a person is willing to spend on a single commuting trip. There are several explanations for the key role it plays in the model. First, instead of Euclidean distances between geographical locations, a more faithful representation of a city's geometry is the set of real travelling times along the spatially embedded, multi-layered, transportation network between individuals (e.g. [27]). Second, there is increasing evidence that the relevant measure for the formation of social ties is *τ*_max_ rather than the spatial separation between pairs of individuals (see [28] for a critical overview). In particular, it has been shown that in cities across the world with high multi-modal commuting behaviours, there is a uniformity in commute times that is independent of travel distance [29].

Here, instead of imposing a single, universal *τ*_max_, such as was done in [18], we allow for a list of different budgets 
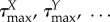
 to reflect the heterogeneity of differing priorities and motivation levels for different activities undertaken by a single, fixed, population. For example, a city dweller who travels for 3 h to attend an important business meeting might not be willing to spend more than 10 min on a weekly drive to a supermarket.

This principle gives us a necessary condition for the existence of a tie:2.3
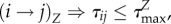
where *τ_ij_* is the travelling-time distance between individuals *i* and *j*.

#### 2.1.4. Intervening opportunities

The fourth principle represents the search heuristic that a person employs to perform constrained optimization and is the defining geometric ingredient of our model. Each potential face-to-face interaction implies a minimal path defined by the shortest connecting travel route, which, in turn, defines a temporal social sphere within which one evaluates the merit of the candidate interaction against other less costly options. These temporal spheres *S_ij_* are simply the sets of people that are closer to individual *i* than another individual *j*, i.e. in a city of population size *N*_pop_,2.4
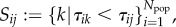
with their cardinalities defining the components of the rank matrix^1^2.5
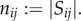


Then, we can express a third necessary condition for a directed social tie as2.6
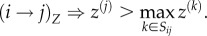


In studies of human mobility, the consideration of such intervening opportunities has been shown to be the key to understanding travel patterns between cities [30,31]. This fourth principle of our model is entirely consistent with and supports the growing body of evidence linking mobility and social contact patterns in cities [24].

As will be shown in the next section, these four principles, together with an assumption or prior knowledge of the spatial distribution of attribute values among the population, are sufficient to construct a weighted, directed network with the nodes {*i*, *j*, …} representing a city's inhabitants and edge weights 
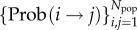
 representing the probabilities of social ties between individuals. This probability network encapsulates the different levels of heterogeneity (attributes, geometry, topology, transport modality and spatial population distribution) in our model of a city. From this probability network, one can extract a host of statistics relevant to the problem at hand. Below we focus on the expected degree, i.e. the expected number of social ties of individuals in a city, which we take as a first measure of *connectivity*, and which turns out to be a strong predictor for several urban indicators.

### 2.2. Counting social ties

By design of the model, the three conditions (2.2), (2.3) and (2.6) are together sufficient for the formation of the social tie (*i*→*j*)*z*. The probability Prob(*i* → *j*)*z* is, therefore, simply the probability that those three conditions are satisfied.

We begin by setting *τ*_max_ → ∞, before reintroducing a finite *τ*_max_ at a later stage. Then by similar reasoning behind the radiation mobility model [30], we have2.7
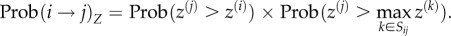


As we show in the electronic supplementary material, S1–S5, this equation can be simplified to give2.8
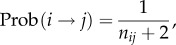
i.e. in the absence of travelling time budget constraints, the probability of a social tie is entirely determined by the rank matrix *n_ij_* (2.5), and is the same for all attributes (hence the dropped *Z* label).

This probability expression is, for large *n_ij_*, virtually equivalent to the proposal Prob(*i* → *j*) = 1/*n_ij_* as introduced in [25] and developed in [18]. Crucially, however, we have shown that it can in fact be derived directly from first principles and is naturally regularized by being well defined when *n_ij_* = 0 without the need for artificial and arbitrarily imposed constraints on the minimum sizes of social spheres [18]. Remarkably also, the attribute-dependency retained at the beginning of our derivation drops out naturally from the final expression—our model is, therefore, a non-trivial instance of a probabilistic and mechanistic social interaction model consistent with observations of emergent urban-feature independence [17].

Clearly, the key input of the model is, then, the travelling-time distance matrix *τ_ij_* from which one uses to build the rank matrix *n_ij_*. The data required for constructing *τ_ij_* are often public and readily available online through a variety of tools,^2^ as demonstrated in the application examples in §4.

The expected total number of ties *T_Z_* corresponding to an attribute *Z* in a population of size *N*_pop_ is then simply the sum over each individual set of probabilities up to a finite 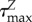, i.e.2.9
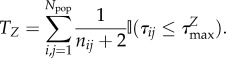


Although technically correct, building the distance matrix *τ_ij_* covering the entire population is highly impractical for all but the smallest of cities. Instead, we subsample the geographical extent of the city at 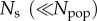 points to generate the much smaller sample distance matrix 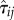. From this coarse-grained representation of the city, we obtain the approximation2.10
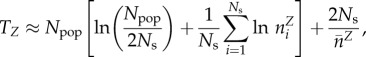
where 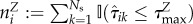 is the size of the social sphere, as related to attribute *Z*, of the location *i* in the subsampled city and 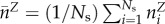 (see the electronic supplementary material for the derivation of this approximation). In the following section, we show through a series of simulations that this approximation is both unbiased and robust.

For the remainder of the paper, we drop the *Z* label for notational clarity.

### 2.3. Local connectivity

The total number of ties *T* is a global, city-wide, connectivity measure which encapsulates the intricate complexities of the city geometry and heterogeneities in agent attributes. Our model also offers a measure that captures the spatial variation in tie-formation across a city. We introduce the concept of the local connectivity of some sub-region of a city as the sum of all incoming and outgoing ties. Let *T_i_* represent the local connectivity at the location of individual *i*, such that 
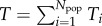
. Then2.11
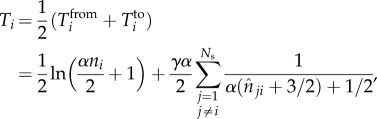
where 
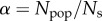
 and *γ* is a scaling factor that ensures, for consistency, that 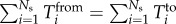 (for a full derivation see the electronic supplementary material).

The distribution of *T_i_* reflects the heterogeneity of the induced interaction network (see electronic supplementary material, figure S3d). In particular, it enables one to quantify the distinct and disproportionate influence that transportation and other infrastructure schemes can have in different parts of the city, as we show in an example in §4.2.

### 2.4. Relating social-tie connectivity with other measurable indicators

Our underlying assumption is that there is a link between the attribute-specific social-tie connectivity *T*, as defined in (2.10), and a measure *U* of a related productive urban activity:2.12
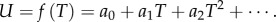


*U* can correspond to socio-economic measures such as gross domestic product (GDP), innovation indices, etc. We are primarily interested here in scenarios where the contribution of individual, isolated, efforts is either non-existent (e.g. spreading of disease) or negligibly small (e.g. collaborative scientific research output). In such cases, *a*_0_ = 0. As a first approximation, we consider here a simple proportional relation with *a_i_*_>1_ = 0, which often provides reasonably good explicative power [18,32]. For example, if the probability *p* of disease transmission in a single encounter between an infected and susceptible individual is small (e.g. sexual per-act human immunodeficiency virus (HIV) transmission risk is less than 0.014 [33]), then within a relatively short timeframe the total number of new infection cases given *T* such interactions is simply *pT*. We, therefore, define our relation to be simply2.13
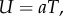
with 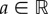 the single unknown parameter relating connectivity and its related activity measure. In situations where the first-order approximation breaks down, the networks of social ties generated through our model allow the use of higher statistics beyond the average degree, which could be used to test hypotheses against (2.12). We discuss this point further at the end of the paper (see also the electronic supplementary material where we discuss the expected degree distribution).

In summary, there are just two parameters in the model: the constant of proportionality *a* and, implicit in the computation of *T*, the travelling-time budget *τ*_max_. We emphasize that these parameters have precise meanings in the model, i.e. they are not just *post hoc* adjustable tuning levers, and that they can be inferred from data to characterize the dynamics and the implications of human interactions contained in the observations (for an example, see §3.4). Alternatively, the parameters, *τ*_max_ in particular, can be fixed using prior knowledge, such as from travel behaviour surveys, information from similar cities or from crowd-sourced location data. Furthermore, under the linear assumption, the typical exercise of comparing scenarios (e.g. the relative increase of economic activity before and after the completion of a new railway) affords a further simplification, as the parameter *a* cancels out when taking ratios.

## 3. Validation of the social-tie model

The mathematical model above formalizes the hypothesis-driven narrative stemming from our set of agent-driven, behavioural principles and represents a possible mechanistic process of face-to-face communication within a general population together with its city-level phenomenological implications. To check the implications of the model, we have performed a set of simulations and empirical validations.

We begin by validating the procedure to obtain *T*, the total number of ties. There are two separate aspects to consider: (i) the statistical validity of the sampling approximation (2.10) for the population-level *T* and (ii) the validity of the rank-based formula (2.8) for the probability of a tie between two individuals given the four principles in our model. We examine both parts together in a single set of simulations, as described below.

### 3.1. Statistical surrogates of cities with multi-modality mobility

To test our model, we generate multiple surrogates of cities and the corresponding travelling-time matrices under multi-modal transport networks. These simulated cities are designed to model real-world urban mobility patterns involving multiple transport modes. We consider four population sizes *N*_pop_ = (300, 500, 800, 1200), with five different population distributions (a uniform distribution over a 45 × 45 km square area, and a two-dimensional, circularly symmetric, Gaussian distribution with standard deviations of 3, 6, 9 and 12 km) and two travelling time budgets (*τ*_max_ = 1, 2 h).

To simulate the multi-modal transportation infrastructure we proceed as follows. For each pair of individuals *i*, *j* in our simulated city, we compute the Euclidean spatial distance *s_ij_* and decompose into binary form3.1
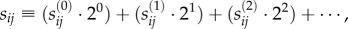
where 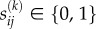. The multi-modality transport network is represented by a speed vector 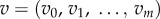, where each component is the speed of a certain transportation mode in order of increasing speed, 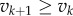. We then generate the travelling-time distance matrix *τ_ij_* between all pairs of points in the city as3.2
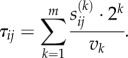


**Figure 1.**
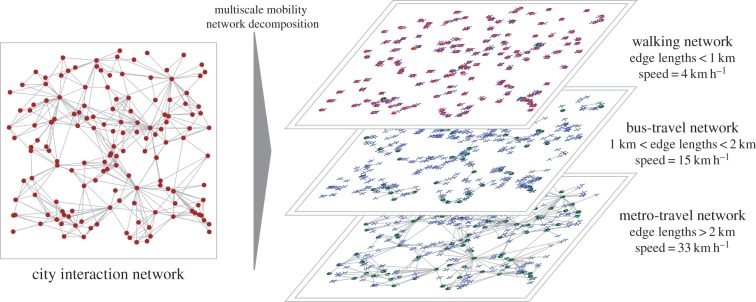
Multi-level mobility network decomposition of urban interaction networks. In the multilayer mobility networks, the red and green nodes represent the origin and destination, respectively, of the particular directed edge in the city interaction network. The blue crosses indicate a transfer from one transport mode to another (e.g. walking to metro), where each cross on a given layer corresponds to another on a different layer. Note that the spatial position of each transfer node in each layer has no meaning other than to provide an indication of the spatial distance travelled in the corresponding mode.

This framework for the simulation of travelling times replicates two features of modern-day transport infrastructure, which is illustrated in figure 1. First, there is the hierarchical nature of travelling speeds with faster transport modes covering larger distances. Second, the framework allows for the fact that travel between two locations in a city typically involves a combination of transport modes (e.g. bus + train). The slowest mode of transportation is given by *v*_0_ = 4 km h^−1^. A city with no transport infrastructure will be represented by a vector **v** = (4, … , 4) and the time between nodes is then the time taken to walk the spatial separation distance. A more realistic case, where public transportation modes of walking, bus and train networks are considered, is represented by **v** = (4, 10, … , 100). If private travel is considered, different classes of roads and expressways traversed using bicycles or automobiles could be considered. In our simulations, we considered four different transport infrastructures, as shown in table 1.

**Table 1.** Travel speeds of four increasingly developed transport infrastructures. **v**^(0)^ represents the trivial case (i.e. no infrastructure). The units are kilometres per hour.

**v**^(0)^ = (4.0, 4.0, 4.0, 4.0, 4.0, 4.0, 4.0, 4.0)
**v**^(1)^ = (4.0, 4.8, 5.8, 6.9, 8.3, 10.0, 11.9, 14.3)
**v**^(2)^ = (4.0, 5.6, 7.8, 11.0, 15.4, 21.5, 30.1, 42.2)
**v**^(3)^ = (4.0, 6.4, 10.2, 16.4, 26.2, 41.9, 67.1, 107.4)

In summary, four population sizes, five distributions, two travelling time budgets, and three non-trivial transportation infrastructures give a total of 120 unique surrogate cities, each given by its specified distribution of *N*_pop_ points on a square 45 × 45 km grid and a resulting *N*_pop_ × *N*_pop_ travelling-time distance matrix *τ_ij_*.

### 3.2. Validation of the sampling procedure and probability model

To validate our sampling (2.10), we compare the travelling-time distance matrix (3.2) in our simulated cities obtained from the whole population *N*_pop_ and from a reduced sample of *N*_s_ = 150 points, as follows. Every one of the 150 × 149 = 22 350 possible directed ties in the sample is assigned a probability according to (2.8). The total number of ties in the sample is obtained by summing over the probabilities, which are then scaled up according to (2.10).

In the simulation of the full population *N*_pop_, we take the viewpoint of each individual, and we rank the other *N*_pop_−1 people in the population according to their travelling-time distances from the individual. We consider a population characterized by an attribute, and the individuals are independent and identically distributed instances drawn from a standard log-normal distribution. There are *N*_pop_(*N*_pop_−1) possible directed ties. Starting from the closest person, a directed tie from the individual is assigned according to the fourth modelling principle of intervening opportunities subject to the upper constraint of an upper bound *τ*_max_ for the travelling time.

**Figure 2.**
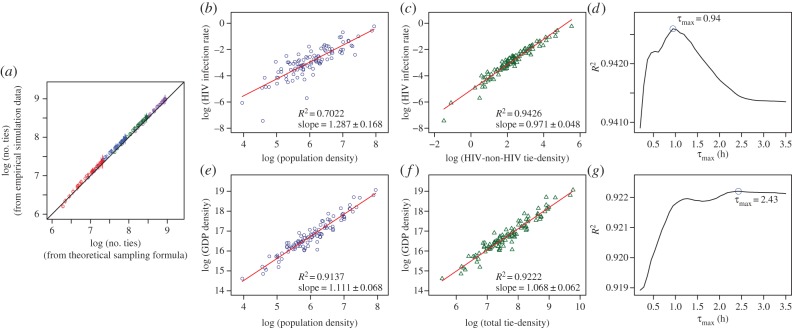
Validation of sampling procedure and empirical validation with HIV infection rates and GDP of 102 US Metropolitan Statistical Areas. (*a*) Comparison of the total number of ties empirically counted according to the interaction model (*y*-axis), with the number of ties estimated from population samples of 120 simulated cities, according to (2.10) and (2.8) (*x*-axis). The four colours (red, blue, green and purple) indicate population sizes of 300, 500, 800 and 1200, respectively. Further variations in the cities are created by imposing different population distributions, maximum travelling-time budgets and transport infrastructure. The circles indicate the mean of 30 simulations and the vertical lines ±2 s.d. As shown, the sampling procedure provides a reasonably good estimate of the total number of ties. (*b*,*e*) Power-law fits of urban indicators to population density. (*c*,*f*) Linear fits of urban indicators to tie-density with *τ*_max_ set at the maximum-likelihood values (as indicated by the blue circles in *d*,*g*). (*d*,*g*) Coefficient of determination of tie-density fits as a function of maximum travelling-time budget *τ*_max_. The error values on the slope parameters indicate ±2 s.d. We note that for both urban indicators, the fits to total tie-density outperform the fits to population density.

The results of the comparison between the full population and the sample are shown in figure 2*a* and the close match demonstrates the validity of the probability model (2.8) as well as demonstrating that the sampling procedure (2.10) provides a good and unbiased approximation.

### 3.3. Comparison with power-law scaling models

Using real-world data from US cities, we compare the predictive abilities of our model and that of power-law scaling models [17]. We begin by generating travelling-time distance matrices on sampled representations of 102 US Metropolitan Statistical Areas (MSAs). The detailed information available^3^ on the population distributions in these MSAs allows us to construct sample distance matrices that are representative of the full population-scale distance matrices. We then plot the computed number of social ties *T* (as a function of the travelling-time budget *τ*_max_) from our model against two measures of urban activity *U*: the 2011 GDP and HIV infection rate.^4^ We also make the comparison with the corresponding power laws against population density. As shown in figure 2, the model is, on its own, well supported by the data with a linear log *U–*log *T* relationship with slope ≈ 1. Our social-tie model provides an equally good fit for the GDP case (*R*^2^ = 0.92 (social ties) versus 0.91 (power law)) and has a significantly stronger statistical support compared to the power-law fit to population density in the HIV infection rate case (*R*^2^ = 0.94 versus 0.70). Much of this improvement stems from the shift from counting people to counting ties—specifically ties between HIV-positive and negative individuals (see the electronic supplementary material). It is the overly broad category of a city's economic output and the lack of specificity in the nature of such relationships that explain the relatively marginal improvement in statistical support in the GDP example. Together, the examples support the view that the fundamental units of a city are not its inhabitants but the social relationships that exist between them.

### 3.4. Evidence for the attribute-dependence of the travelling-time budget

In addition to its predictive performance shown above, and because of its agent-driven construction, our model can also shed light on the mechanistic origin of social interactions. For instance, the two examples above (GDP and HIV infection) highlight a marked difference in the underlying social dynamics across the two attributes considered, as seen from the corresponding maximum-likelihood estimates of *τ*_max_. We obtain *τ*_max_ = 2.43 h (95% CI [0.36 h, 5.42 h]) for the GDP output versus a markedly lower value of *τ*_max_ = 0.94 h (95% CI [0.36 h, 1.52 h]) for HIV infection rates. The confidence intervals are given by quantiles from bootstrapped samples of the original dataset (see the electronic supplementary material).

Ignoring for the moment the small range of variation in *R*^2^ values with *τ*_max_, there are two immediate interpretations. First, our fits indicate that, in contrast to economically productive activities, it is unlikely that one would be willing to travel for more than 1.5 h to engage in activities associated with HIV transmission. Second, as expected, GDP stems from a wide range of activities leading to a more variable *τ*_max_. Recognizing and quantifying such differences in interpretable parameters and their variances, which would be missed by simple scaling arguments, is of relevance in efforts to build both prosperous and healthy cities.

Nevertheless, despite the bootstrapped analysis giving confidence intervals for our *τ*_max_ estimates, the small range of variation in *R*^2^ suggests a level of redundancy in our model with the constant of proportionality *a* in (2.13) affording too much freedom. In order to increase the robustness of the model when applied to real data, we eliminate the proportionality parameter *a* by considering relative increases of indicators, i.e. we consider the ratio *U*_1_/*U*_2_ of the economic indicators. This is illustrated in the next section, where we provide two examples of the application of this approach.

## 4. Applications of the social-tie model

To illustrate the applicability of our model, we examine two examples of large-scale transportation projects in the UK: High Speed 2 (HS2) and London Crossrail.

### 4.1. The High Speed 2 project

**Figure 3.**
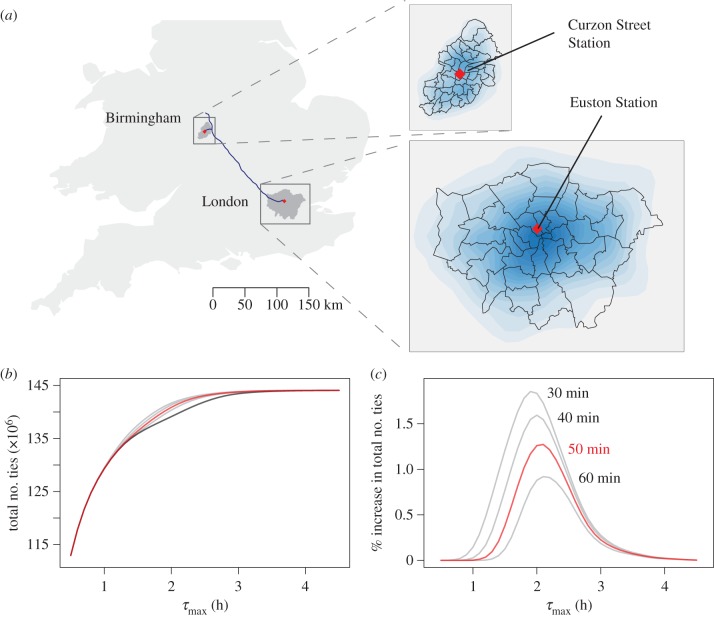
High Speed 2 (Phase 1) and its impact on the connectivity of UK cities. (*a*) HS2 (Phase 1) route and the population densities of London and Birmingham. The blue line indicates the published proposed route of the first phase of HS2 (as of December 2013). The red diamonds indicate the locations of the rail stations in each city. The contour maps are derived from kernel density estimates of 1000 and 129 sample points in London and Birmingham, respectively. The ratio of the number of samples is chosen to reflect the relative sizes of the two cities. (*b,c*) Impact of HS2 (Phase 1) on the connectivity of UK cities. The black curve indicates the connectivity without HS2. The red curves indicate the connectivity according to the planned improved travel times (50 min between London and Birmingham). The grey curves in (*c*) indicate hypothetical travel times of 30, 40 and 60 min.

HS2 is the proposed high-speed rail network connecting the major cities in Britain, from London in the south to the northern cities of Leeds, Manchester and beyond. In this section, we focus on the first-phase link between London and Birmingham that would reduce the one-way travel time from the current 84 to 50 min. We treat the two cities as a single conurbation and omit the influence of the neighbouring regions; the results presented here should be interpreted in the light of this geographical treatment. In figure 3, we plot the total and percentage increases in the number of ties as a function of *τ*_max_. If we take the value of *τ*_max_ = 2.43 h, which we inferred previously for the GDP-related travelling-time budget, the average economic boost induced by the presence of HS2 across the two cities would be ≈0.96%. A more robust approach is to consider a range of possible time budgets to evaluate the effect of uncertainty in *τ*_max_ (see the electronic supplementary material). For instance, assuming a uniform distribution over 1 < *τ*_max_ < 3, we obtain an increase in GDP of 0.80%. Interestingly, we observe a middle ‘sweet spot’ at *τ*_max_ ∼ 2 h: at the lower tail, the journey times are insufficiently short to tempt one to travel further, while at the upper tail, the efforts are wasted on a population already willing to endure long commutes.

### 4.2. London Crossrail

**Figure 4.**
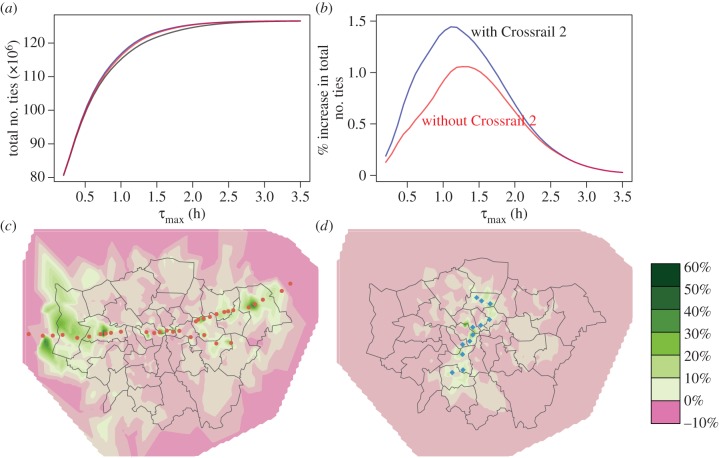
Impact of London Crossrail on city-wide and local connectivities. (*a,b*) Impact of London Crossrail on the connectivity of London. The black curve indicates the present connectivity without Crossrail. The red curves indicate the connectivities according to the planned improved travel times from Crossrail (but without Crossrail 2). The blue curve in (*b*) shows the connectivity boost by including Crossrail 2 (metro-only option), a proposed project extension to include a north–south train link. (*c*) Percentage change in local connectivity due to Crossrail. (*d*) Percentage change in local connectivity due to Crossrail 2 (metro-only option) relative to post-Crossrail. The heat map scales indicate percentage change in the total number of incoming and outgoing ties for each region. The red points indicate the Crossrail stations and the blue points the 12 proposed Crossrail 2 stations.

Crossrail is a high-frequency railway linking east and west London currently under construction. Under the same *τ*_max_ assumptions as for HS2 above, the projected impact of Crossrail on the London economy is a 0.3% increase in the city's GDP (with an increase of 0.61% for the uniform distribution of *τ*_max_; figure 4). The percentage increases may appear small (less than 1%), but are by no means unexpected for two reasons. First, the stated investment cost is itself a small fraction of London's GDP. Second, the modest boost is simply a reflection of the highly concentrated population density in the central regions and the extensive transport infrastructure already in place.

The availability of precise local geographical data allows us to further interrogate the model to determine the spatial distribution of local connectivities *T_i_* (2.^11^). Indeed, it is important to note that neither the current local connectivity levels nor the impact of Crossrail are evenly distributed or felt across the city (figure 4). As would be expected, the largest increases are found near railway stations, especially in London's suburbs. As we explore further (see electronic supplementary material, figure S5), there is a concentration of newly possible connections along the east–west extent of the city. More surprisingly, however, we observe a *decrease* across large areas along the orthogonal north–south axis driven by falls in their *relative* accessibility—the rising tide of connectivity does not lift all boats. This effect may be unavoidable, but the ability to quantify and map its spatial extent allows one to anticipate and, possibly, alleviate its impact.

There is a mooted north–south extension—*Crossrail 2*—which is currently under study (see the electronic supplementary material for details). In similar fashion to Crossrail, the expected additional boost to GDP can be calculated and is shown in figure 4. Crucially, in line with one's intuition, the negative local impact is now distributed outside the areas surrounding the Crossrail 2 rail line.

## 5. Discussion

Unlike typical social network and epidemiological studies that assume a fixed and known network structure within which various dynamical processes (e.g. spread of diseases) are constrained, our approach obtains interaction networks as induced structures that emerge from the application of our set of principles to different cities. In this sense, these interaction networks are unobserved structures, much like genealogical trees in population genetics [34]. Unlike random geometric graphs emerging in models of cities with uniform population distributions [35], our model incorporates agent-driven optimization principles and physical constraints from the geometry and topology of each city. Hence, rather than functioning as input features for our model, these resulting networks capture and are confined by the make-up of the demographic and transport infrastructure data under study.

Although the unobservable nature of the underlying connectivity networks poses challenges for the direct validation of our model, the recent availability of large-scale location data from mobile phones appears to offer a wealth of possibilities for testing some of the model assumptions, e.g. the existence of travelling-time budgets 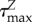, and their assumed uniformity across the population for each attribute. However, there are specific conditions that such empirical studies must fulfil. In particular, one should be able to identify, with reasonable certainty, the purpose and deliberateness of both single journeys and social ties observed. In this context, the growth of location-based and, crucially, activity-specific, social networking services could provide valuable information [36], in contrast to simply relying on proximity information for social tie prediction [37].

As shown above, the overall connectivity *T* is, on its own, a strong predictor for several urban indicators and we have concentrated on this aspect in this paper. This is reassuring given the known ability of mean-field theory to capture basic trends [38] on networks. Nevertheless, further details and statistics (e.g. heterogeneity) of the obtained networks could be studied, as the mechanistic and constructive nature of our model provides the necessary information for extracting these additional features. We provide a short illustration of this process in the electronic supplementary material. An extension of our model will be to propose and test the analogue of (2.13) with different network statistical measures in place of *T*.

The generic nature of the proposed framework and the increasing availability of geo-location and travel data ensure a broad and growing array of applications. This includes gauging the robustness of a city to traffic congestions and measuring the cost of weather-related disruptions. Methodological extensions to the model might include, for instance, replacing travel time with a cost function incorporating spatial distance, financial cost and the time of day.

Our focus for most of this paper has been on the city as defined by civil administrative conventions. Since studies of cities are sensitive to the exact definition of a city itself [39,40], there is the option of adopting one of the more nuanced alternative definitions that do not include any arbitrary geographical boundaries [41]. However, the model itself is actually agnostic as to the source of the population variables *N*_pop_ or the travelling-time distance matrices *τ_ij_*, as indeed we have shown by treating the two cities of London and Birmingham as a single entity in our analysis above. Our approach can thus be applied to reflect the connectivity among geographical entities both on a larger scale (countries or larger geographical regions) and a smaller scale (buildings or campuses). On such smaller scales, this approach can inform design to maximize the creative, social and economic benefits resulting from human encounters. Regardless of the context of application, it is not the actual spatial size but the extent *perceived* via travelling times that determines the connectivity of a system. Large cities may be great, but great cities most certainly look small.

## Supplementary Material

Supplementary Information

## Endnotes

1 Note that, in general, *n_ij_* ≠ *n_ji_*.

2 For example, Google Distance Matrix API, MapQuest Route Matrix, Microsoft Bing Routes API.

3 *2010 Census of Population and Housing* and *2010 U.S. Metropolitan Statistical Area Distance Profiles*, www.census.gov; www.microsoft.com/maps/.

4 US Centers for Disease Control and Prevention. *HIV Surveillence Report*, 2011; vol. 23. www.cdc.gov/hiv/topics/surveillance/resources/reports/. Feb. 2013.
