# Supplementary material for: Great cities look small
Source: J R Soc Interface. 2015 Aug 6;12(109):20150315. doi: 10.1098/rsif.2015.0315 (PMC4535402; doi:10.1098/rsif.2015.0315)
Supplement: Supplementary Information [file rsif20150315supp1.pdf]

Great cities look small – Supplementary Material

Aaron Sim
Department of Mathematics &
Department of Life Sciences,
Imperial College London, SW7 2AZ, UK

Sophia N. Yaliraki
Department of Chemistry,
Imperial College London, SW7 2AZ, UK

Mauricio Barahona*
Department of Mathematics,
Imperial College London, SW7 2AZ, UK

Michael P. H. Stumpf†
Department of Life Sciences,
Imperial College London, SW7 2AZ, UK

June 19, 2015

S1 Derivation of social-tie formulae

S1.1 Rank-based tie probability

Let $\{Z^{(i)}\}_{i=1}^{N_{\rm pop}}$ be a set of positive real-valued random variables representing a single attribute for individuals in a population of size $N_{\rm pop}$. We assume that the random variables are independent and identically distributed (i.i.d.) according to some distribution $q(z|z \in \mathbb{R}^+)$. Let τ_{ij} be the distance matrix specifying the travelling-time distances between individuals, and S_{ij} the temporal social-spheres given by the sets

$$S_{ij} := \{k \,|\, \tau_{ik} < \tau_{ij}\}, \tag{S1}$$

The number of people that are closer to individual i than j as determined from τ_{ij} is represented by the rank matrix n_{ij}, which is simply the cardinality of the temporal spheres, i.e.

$$n_{ij} := |S_{ij}|. \tag{S2}$$

By design of the proposed social interaction model, in the case where $\tau_{\rm max} \to \infty$, a directed tie from i to j is formed if and only if,

$$z^{(j)} > z^{(i)} \qquad \text{and} \qquad z^{(j)} > \max_{k \in S_{ij}} z^{(k)}, \tag{S3}$$

where $z^{(i)}$ is a realisation of $Z^{(i)}$ and S_{ij}. Let z_n be the maximum value obtained from m random samples from $q(z)$, and let $P_n(\mathrm{c})$ be the probability that z_n satisfies some condition c. Then, from (S3), the probability of a directed tie from individual i to j is

$$\begin{aligned}\mathrm{Prob}(i \to j) &= \mathrm{Prob}(z^{(j)} > z^{(i)}) \times \int_0^\infty P_1(> z) P_{n_{ij}}(= z)\,\mathrm{d}z \\ &\equiv \int_0^\infty P_1(> z) P_{n_{ij}+1}(= z)\,\mathrm{d}z.\end{aligned} \tag{S4}$$

Since the population individuals are assumed to be i.i.d. w.r.t. $q(z)$ we have

$$P_1(> z) = 1 - q(< z), \tag{S5a}$$

$$P_{n_{ij}+1}(= z) \equiv \frac{\mathrm{d}P_{n_{ij}+1}(< z)}{\mathrm{d}z} = (n_{ij}+1)\big[q(< z)\big]^{n_{ij}}. \tag{S5b}$$

Substituting (S5) into (S4) and changing variables gives

$$\begin{aligned}\mathrm{Prob}(i \to j) &= \int_0^1 \big[q(< z)^{n_{ij}} - q(< z)^{n_{ij}+1}\big]\,\mathrm{d}q(< z) \\ &= \frac{1}{(n_{ij}+2)}.\end{aligned} \tag{S6}$$

This is Eq. (8) in the main text. Remarkably, this is independent of the specific attribute Z under consideration. For large rank n_{ij} and up to a constant of proportionality, this expression for the tie-formation probability closely resembles the original rank-based ansatz $\mathrm{Prob}(i \to j) = 1/n_{ij}$ in [1, 2]. The difference here is that the attribute-independence of the tie-probability is an emergent feature rather than a theoretically unsupported assumption of universality.

S1.2 Social-tie sampling approximation

Following [1] we first assume a uniform population density ρ and travelling-time budget $\tau_{\rm max}$. The density is

*m.barahona@imperial.ac.uk
†m.stumpf@imperial.ac.uk

measured in terms of number of individuals per travelling time 'volume'. The number of ties $t_i(\rho)$ to a given individual i is

$$t_i(\rho) = \int_0^{\tau_{\max}} \frac{2\pi\tau\rho\,\mathrm{d}\tau}{\rho\pi\tau^2 + 2}\,\mathrm{d}\tau = \ln(\rho\pi\tau_{\max}^2 + 2) - \ln 2 = \ln\left(\frac{S_i}{2} + 1\right), \tag{S7}$$

with $S_i = \rho\pi\tau_{\max}^2$ the size of the temporal social-sphere, i.e. number of nodes reachable from node i. Here we are first evaluating the probability of an individual at the origin finding another individual with higher attribute value in a differential spherical volume with the radius is given by the minimum travelling-time distance on the underlying network. Expanding the radius of action on the network, we can then geometrically determine the expected number of ties by integrating up to an attribute-specific limit.

We now drop the dependence on the constant uniform density ρ where the allowance for a heterogeneous distribution is reflected in a varying S_i for different nodes i. We replace $t_i(\rho)$ with t_i to indicate this transition. The total number of ties T in the population is, then, simply

$$T = \sum_{i=1}^{N_{\mathrm{pop}}} t_i = N_{\mathrm{pop}}\left[\frac{1}{N_{\mathrm{pop}}}\sum_{i=1}^{N_{\mathrm{pop}}} \ln\left(\frac{S_i}{2} + 1\right)\right] \le N_{\mathrm{pop}} \ln\left[\frac{1}{N_{\mathrm{pop}}}\sum_{i=1}^{N_{\mathrm{pop}}}\left(\frac{S_i}{2} + 1\right)\right] = N_{\mathrm{pop}} \ln\left(\frac{\bar{S}}{2} + 1\right), \tag{S8}$$

where $\bar{S} = 1/N_{\mathrm{pop}} \sum_{i=1}^{N_{\mathrm{pop}}} S_i$ is the population average of the number of reachable nodes. The inequality is due to Jensen's inequality and the concavity of the logarithmic function.

However obtaining the full set $\{t_i\}_{i=1}^{N_{\mathrm{pop}}}$ is neither possible or practical for typically-sized cities. We therefore take a sample of N_s points. Defining $\alpha \equiv N_{\mathrm{pop}}/N_s$, if the sample is representative of the population, we have

$$\frac{\bar{S}}{\bar{n}} = \alpha, \tag{S9}$$

where $\bar{n}$ is the average of the number of reachable nodes within the sample set, i.e. $\bar{n} = (1/N_s)\sum_{i=1}^{N_s} n_i$. We have

$$\begin{aligned} N_{\mathrm{pop}} \ln\left(\frac{\bar{S}}{2} + 1\right) &= N_{\mathrm{pop}} \ln\left(\frac{\alpha\bar{n}}{2} + 1\right) \\ &= N_{\mathrm{pop}} \ln\left[\frac{\alpha\bar{n}}{2}\left(1 + \frac{2}{\alpha\bar{n}}\right)\right] \\ &= N_{\mathrm{pop}}\left[\ln\frac{\alpha}{2} + \ln\bar{n} + \ln\left(1 + \frac{2}{\alpha\bar{n}}\right)\right] \\ &\approx N_{\mathrm{pop}}\left(\ln\frac{\alpha}{2} + \ln\bar{n} + \frac{2}{\alpha\bar{n}}\right) \\ &\ge N_{\mathrm{pop}}\left[\ln\frac{\alpha}{2} + \frac{1}{N_s}\sum_{i=1}^{N_s}\ln n_i + \left(\frac{2}{\alpha\bar{n}}\right)\right]. \end{aligned} \tag{S10}$$

Combining (S8) and (S10) we expect the two inequalities to cancel out approximately, giving

$$T \approx N_{\mathrm{pop}}\left[\ln\left(\frac{N_{\mathrm{pop}}}{2N_s}\right) + \frac{1}{N_s}\sum_{i=1}^{N_s}(\ln n_i)\right] + \frac{2N_s}{\bar{n}}. \tag{S11}$$

This is Eq. (10) in the main text.

S1.3 Local connectivity

The local connectivity is defined as half the sum of incoming and outgoing ties from a given location. Let T_i represent the local connectivity of the location of individual i. By definition, we have

$$T_i = \frac{1}{2}\left(T_i^{\mathrm{from}} + T_i^{\mathrm{to}}\right), \qquad \text{with} \qquad \sum_{i=1}^{N_{\mathrm{pop}}} T_i = T. \tag{S12}$$

As in the case of global connectivity, the key is to approximate T_i without having access to the population distance matrix. We estimate the outgoing and incoming contribution separately, beginning with the outgoing component T_i^{from}.

Following the reasoning behind (S10), we have

$$T_i^{\mathrm{from}} = \frac{1}{2}\ln\left(\frac{\alpha n_i}{2} + 1\right). \tag{S13}$$

Quantifying the incoming ties is less straightforward as there is no simple scaling from the sample. Instead we perform the approximation in three stages on the basis of several reasonable assumptions. First, for two individuals i and j in our population sample, we approximate the true population rank n_{ij} from the sample rank $\hat{n}_{ij}$, i.e.

$$n_{ji} = (\alpha - 1) + \frac{1}{2}(\alpha - 1) + \alpha\hat{n}_{ji} = \alpha\left(n_{ji} + \frac{3}{2}\right) - \frac{3}{2}, \tag{S14}$$

where the three terms in the sum are, respectively, the scaled contributions from individuals i, j and the $\hat{n}_{ji}$

intervening samples[1]. Second, from (S6) and (S14), the probability of an directed tie from j to i is

$$\text{Prob}(j \to i) = \frac{1}{\alpha(n_{ji} + \frac{3}{2}) + \frac{1}{2}}. \tag{S15}$$

A first approximation of the expected total incoming ties is the appropriately-scaled sum of all incoming tie probabilities from our sample, i.e.

$$T_i^{\text{to}} = \alpha \sum_{\substack{j=1\\ j\neq i}}^{N_s} \text{Prob}(j \to i). \tag{S16}$$

However, imposing the consistency criteria $\sum_{i=1}^{N_s} T_i^{\text{from}} = \sum_{i=1}^{N_s} T_i^{\text{to}}$ requires a third step of scaling (S15) appropriately. Therefore, we have

$$T_i^{\text{to}} = \gamma\alpha \sum_{\substack{j=1\\ j\neq i}}^{N_s} \text{Prob}(j \to i), \tag{S17}$$

with

$$\gamma = \frac{\sum_{i=1}^{N_s} T_i^{\text{from}}}{\alpha \sum_{i=1}^{N_s} \sum_{j=1,j\neq i}^{N_s} \text{Prob}(j \to i)}. \tag{S18}$$

Substtituing (S13) and (S17) into (S12), we obtain Eq. (11) in the main text.

S2 Induced network structure from social dynamics

In this paper we have constructed a probability model for generating social-tie networks where the edges denote deliberate (i.e. planned as opposed to random encounters) face-to-face interactions. It is worth reemphasising that the networks throughout are themselves unobserved structures, which compels one to average over all possible networks. In this section we provide three instances of how our model can be coaxed to provide additional secondary expected network summary statistics. This is in addition to the expected number of interactions, i.e. the expected number of network edges, which we examined in the section above, and have showed in the main text to be a sufficiently strong predictor for several urban indicators. Specifically, we look at network heterogeneity, multilevel network structures, and spatial extent of spatial networks.

S2.1 Network heterogeneity

We focus on the impact that different population distributions and travelling-time budgets have on the expected network degree distributions. We simulate three cities following the procedure outlined in the section above. The first two cities are networks with 150 nodes uniformly distributed with average $\tau_{\max} = 0.35$ and $\tau_{\max} = 0.35$ respectively, while the third is a network with 150 nodes sampled from a $(1/3, 2/3)$-weighted mixture of a uniform and Gaussian distribution with component standard deviation of 4 km and average $\tau_{\max} = 0.5$. The travelling-time budgets were chosen such that the second and third networks possess a similar number of edges. In both cities the transport infrastructure is assumed to have three modes and is represented by the speed vector

$$v' = (4.0, 15.0, 15.0, 33.0, 33.0, 33.0, 33.0, 33.0). \tag{S19}$$

As before, the values have units of kilometres per hour and here the three values represent the average speeds of walking, bus, and metro travel. Three example networks for a given population distributions of attribute values are shown in Figure S2. First we observe, somewhat trivially, that for a single city an increase in $\tau_{\max}$ can lead to an increase in number of edges. Second, for similar spatial distributions, we see that the network degree distributions of two cities can be markedly different, even in the case where the number of edges are similar. Here we compare the expected degree distribution, taking the average over 120 random attribute-value ranking distributions. As shown in Figure S2, the city with a dense centre has a significantly higher level of network heterogeneity than the uniformly distributed city.

S2.2 Spatial extent of spatial networks

Since the underlying interaction networks behind the connectivity measure are spatial networks, it can be useful to examine the impact of urban infrastructure changes not just on overall and local connectivities, but on the spatial nature of those changes. In this section we use the example of London Crossrail from the main text. There we calculate both the impact on total connectivity and its local spatial variations. Here, we go one step further by predicting the expected distribution of the interaction network edge lengths in the city of London (in terms of Euclidean spatial distances) before and after the construction of London Crossrail.

The results are presented in Figure S3. We make three observations. First, the newly possible interactions (i.e. those with probability zero in the absence of Crossrail) tend to have higher average edge lengths than existing interactions. Second, the existing connections that are upgraded or downgraded in probability seem to have identical spatial length distribution. Third, the increases tend to occur along the new railway route while the decreasing edges tend to have one or both nodes in the orthogonal dimension (i.e. north-south corridor). The conclusion here is highly intuitive: apart from new connection possibilities between regions that are otherwise separated by large spatial, the changes in interaction probabilities

[1] NB: $n_{ij} \neq n_{ji}$. We assume throughout that individual i is the seeker, i.e. the *recipient* of incoming ties.

depends less of distance between nodes than the nodes' locations relative to the new infrastructure.

S3 Details of empirical validation examples

102 US Metropolitan Statistical Areas (MSAs) were chosen on the basis of the availability of HIV infection rate, GDP, and spatial population distribution data.

S3.1 Data sources

The population statistics and density profiles of the 102 US MSAs are obtained from the U.S. Census Bureau[2]. HIV infection and prevalence data are obtained from the United States Centers for Disease Control and Prevention[3]. Travel times between city locations are obtained using Microsoft BING maps[4] and for car journeys originating at 1200h local-time on 13th Dec 2013.

Given the marginal radial distribution of the population, we assume a circular symmetry about the central city hall location and sample a set of 1000 points for each US MSA. One can drop this assumption and obtain more accurate and precise population distribution data, for instance from detailed local census and other open data sources. The total number of ties T_i in each MSA i is then calculated by applying (S11) to the travelling-time distance matrices obtained from online mapping resources. The mode of transport here is restricted to travel by roads – an assumption that is reasonable for many US MSAs.

In the example of HIV infection rates, the relevant number of ties are encounters, T', between HIV-positive and HIV-negative individuals, rather than the total number of ties. We therefore scale the total no. of ties by the mixed-ties proportions, giving

$$T' = \frac{2H(N-H)}{N(N-1)} T, \tag{S20}$$

where N is the population size of a given MSA and H the number of individuals diagnosed as HIV-positive, which we take to be equal to the reported number of HIV-positive individuals in the population.

S3.2 Robustness of τ_{max} estimation

In this section we gauge the robustness of the maximum likelihood travelling-time budget estimates τ_{max}^{mle} obtained for the HIV-infection rates and GDP-related attributes by constructing confidence intervals (C.I.) around the respective point estimates. We present two versions: a bootstrap C.I. and a C.I. based on the asymptotic variance of the maximum likelihood estimator in terms of the observed Fisher information.

Using $N_B = 1000$ bootstrap replicates of the original set of 102 US cities, we repeat the linear fits of the urban indicators to tie-density. We obtain a set of bootstrap travelling-time maximum likelihood estimates $\{\tau_{\text{max},i}^{mle}\}_{i=1}^{N_B}$ which then provides a bootstrap confidence interval C_{boot} in terms of the empirical quantiles [3].

Next, we assume that the residues of the $\log U - \log T$ linear fit $\log U = g_{\tau_{\text{max}}}(\log T)$ are normally distributed with $\hat{s}$ be the sample standard deviation of the maximum likelihood fit $g_{\tau_{\text{max}}^{mle}}$. We further assume that the data points are independent, whereby the likelihood is

$$\mathcal{L}(\tau_{\text{max}}) = \prod_{i=1}^{n} f_{\tau_{\text{max}}}(\log U_i). \tag{S21}$$

$f_{\tau_{\text{max}}}(\log U_i)$ the univariate normal density function with mean $g_{\tau_{\text{max}}}(\log T_i)$ and variance $(\frac{\hat{s}}{\hat{s}-1})^2$. The observed Fisher information I is then

$$I = -\left.\frac{d^2 L(\tau_{\text{max}})}{d^2 \tau_{\text{max}}}\right|_{\tau_{\text{max}}=\tau_{\text{max}}^{mle}}, \tag{S22}$$

where $L = \log \mathcal{L}$ is the log-likelihood. Practically, we obtain (S22) through a series of Gaussian Process fits though the set of emprical data points $\{\tau_{\text{max}}, L(\tau_{\text{max}})\}$. In the asymptotic limit, the maximum likelihood estimator is normally distributed with variance $-1/I$ which is used to define the C.I. C_{mle}. Strictly speaking, the asymptotic distribution is clearly not normal as the parameter $\tau_{\text{max}} > 0$. However, at least for the GDP attribute, the maximum likelihood estimate is sufficiently away from the zero boundary for this to be a reasonable assumption.

The C.I.s are illustrated in Figure S4 for both attributes. From the analysis we have the 95% C.I. $C_{\text{boot}} = [0.36, 1.52]$ and $C_{\text{boot}} = [0.36, 5.42]$ ($C_{\text{mle}} = [0.15, 4.65]$) for the HIV infection rates and GDP-related attributes respectively. While the τ_{max} estimate for the HIV infection rates attribute is fairly robust, the C.I. for the GDP estimate spans > 4 hours. This behaviour confirms the intuition that the GDP indicator pertains, in reality, to an amalgamation of many attributes with varying sizes of τ_{max}. For instance, a typical city inhabitant is unlikely to patronise a laundromat more than a few minutes from home; on the other hand, the same person is probably willing to endure a long commute across the city for a one-off visit to a unique theme park, say. Both activities contribute to GDP, and this difference is reflected in the wide span for the τ_{max} estimates.

[2] *2010 Census of Population and Housing, 2010 U.S. Metropolitan Statistical Area Distance Profiles*, www.census.gov

[3] US Centers for Disease Control and Prevention. *HIV Surveillance Report*, 2011; vol.23. www.cdc.gov/hiv/topics/surveillance/resources/reports/. Feb 2013.

[4] www.microsoft.com/maps/

S4 Details of HS2 and London Crossrail analysis

S4.1 Data sources

London and Birmingham demographic profiles and geographical details are obtained from the Greater London Authority[5] and the Birmingham City Council[6] respectively. Details of HS2, including routes, station locations and travel speeds are obtained from the High Speed Two Limited[7]. London Crossrail station and travel times are obtained from Transport for London[8]. Current travelling times between city locations are obtained using Microsoft BING maps[9].

We obtain geographic samples from the cities of London and Birmingham, UK from two-dimensional (weighted) kernel density estimates (KDE) of the population spatial distributions. The central locations of the 32 boroughs in London and 40 wards in Birmingham are treated as data points with weights proportional to the local population sizes. We use a Gaussian kernel with bandwidth equal to 1.2 times the radius of a circle with area equal to the local borough or ward for each data point. The population of each city is then sampled from this weighted mixture of Gaussians. We have a total of 1000 and 128 location samples for London and Birmingham respectively.

The travelling time distance matrices used represent, for the majority of point-pairs, public transport travelling time. In the absence of public transport data between two locations, we assume that the relevant journey is taken by car. As for the US MSA examples, the data is obtained from online mapping resources. We selected a departure time of 1200 on 12th December 2013.

For the HS2 example, we assume a single interchange station in each city (London Euston, and Curzon Street station in Birmingham). The travelling time between locations in each city is a sum of the travelling times to each station and the published journey time between the two cities (we do not factor in waiting times, delays, etc.).

There are 36 stations on the London Crossrail network, with an additional 12 in the CrossRail 2 (metro-only option) extension. The improved travelling time between two London locations is the sum of the travelling times from the origin and destinations to their respective closest (by time) Crossrail stations and the published station-to-station journey time.

[5] http://data.london.gov.uk/datastore
[6] www.birmingham.gov.uk
[7] www.hs2.org.uk
[8] www.crossrail.co.uk
[9] www.microsoft.com/maps/

References

[1] Wei Pan, Gourab Ghoshal, Coco Krumme, Manuel Cebrian, and Alex Pentland. Urban characteristics attributable to density-driven tie formation. *Nature Communications*, 4:1961, June 2013.

[2] David Liben-Nowell, Jasmine Novak, Ravi Kumar, Prabhakar Raghavan, and Andrew Tomkins. Geographic routing in social networks. *Proceedings of the National Academy of Sciences of the United States of America*, 102(33):11623–11628, August 2005.

[3] Bradley Efron and Robert J Tibshirani. *An introduction to the bootstrap*, volume 57 of *Monographs on Statistics and Applied Probability.* Chapman and Hall, New York, 1993.

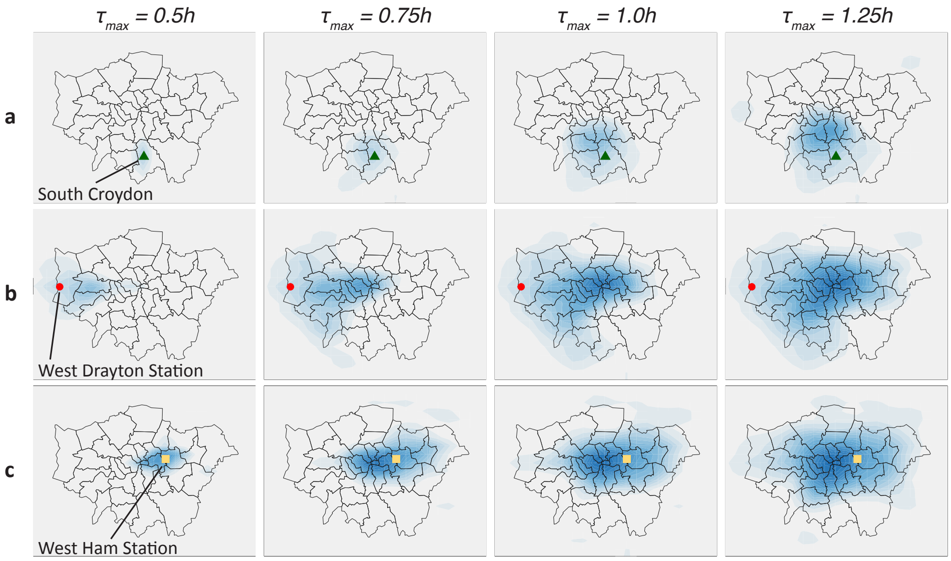

Figure S1: **Density maps of *travelling-time social spheres* in London (with Crossrail) as a function of $\tau_{\mathbf{max}}$ and location.** The coloured square, circle and triangle represent example central, western, and southern locations in London respectively. The contour maps represent kernel density estimates of samples ($N_s = 1000$) within the indicated travelling-time distance budget. The western location in **b** lies directly on a Crossrail station (West Drayton station), while the southern location (South Croydon station) in **c** is chosen to illustrate a relatively inaccessible location in the city. See *SI* Section S4 for details of the construction of the distance matrices used.

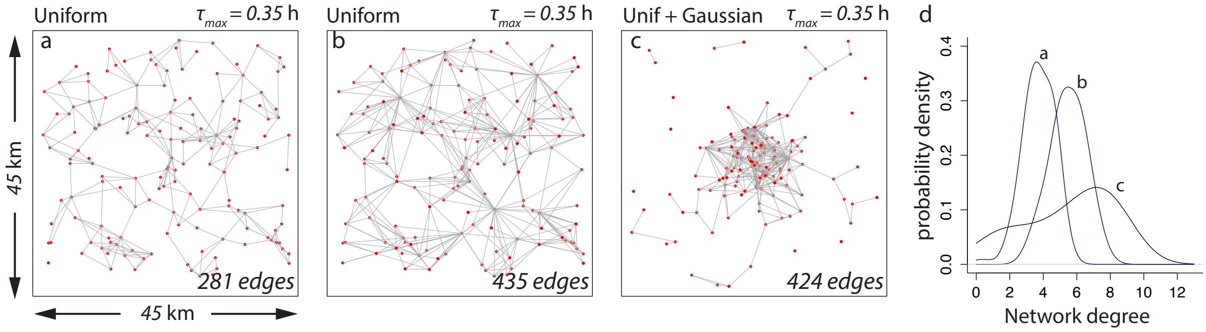

Figure S2: **Emergence of network structure**. **a,b,c,** Simulated city interaction network examples. The red nodes represent individuals while the network edges indicate a directed social-tie (direction not specified). The nodes in networks **a** and **b** are uniformly distributed while those in network **c** is sampled from a (1/3, 2/3)-weighted mixture of a uniform and Gaussian distribution with component standard deviation of 4 km. **d,** The network degree distributions, averaged over 120 different (and random) attribute-value distributions, for the three simulated cities.

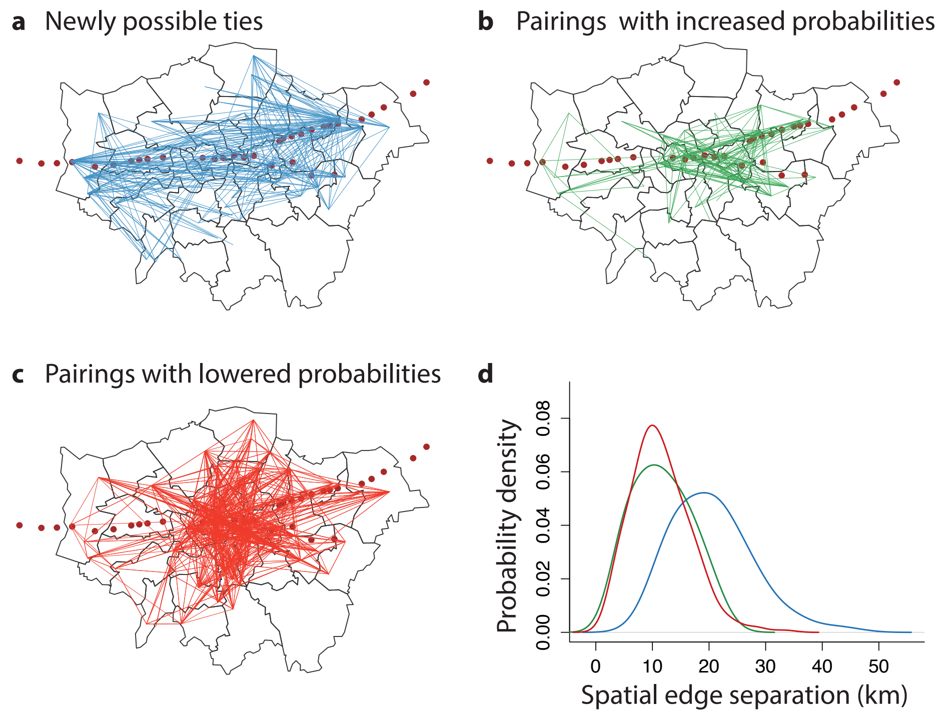

Figure S3: **Crossrail effect on London interaction network**. **a**, Newly possible interaction edges, **b**, existing possible interactions that have increased in probability, **c**, existing possible interactions that have decreased in probability. The three networks are taken from a subnetwork with 70 nodes. **d**, Expected distribution of the interaction network edge lengths for the three classes of interactions. The edge lengths are given in terms of the spatial Euclidean distances between nodes.

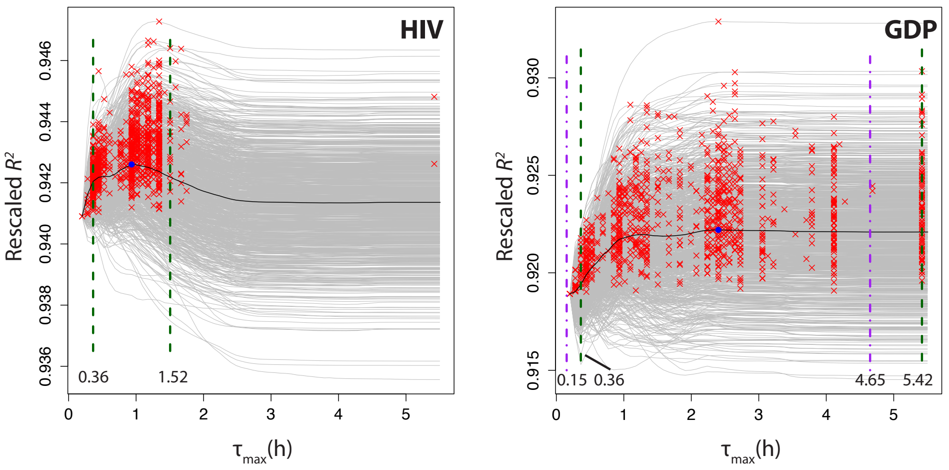

Figure S4: **Robustness of $\tau_{\mathbf{max}}$ estimates**. Plots of rescaled-R^2 values of $\log U - \log T$ linear fits as a function of $\tau_{\max}$. The black solid line is the curve using the original dataset of 102 cities. Each of the 1000 grey curves represents the R^2 values of a separate bootstrap sample of the original data, rescaled such that $\tau_{\max}$ matches the value from the original dataset. The red crosses indicate the maximum R^2 values of the bootstrap curves and the blue circle the same for the original curve. The green dashed and purple dot-dashed lines indicate the 95% bootstrap confidence interval and the observed Fisher information-derived confidence interval respectively.
